# Electrodeposition of Mesoporous Ni-Rich Ni-Pt Films for Highly Efficient Methanol Oxidation

**DOI:** 10.3390/nano10081435

**Published:** 2020-07-23

**Authors:** Raül Artal, Albert Serrà, Johann Michler, Laëtitia Philippe, Elvira Gómez

**Affiliations:** 1 Thin Films and Nanostructures Electrodeposition Group (GE-CPN), Department of Materials Science and Physical Chemistry, University of Barcelona, Martí i Franquès 1, E-08028 Barcelona, Spain; rartal2@gmail.com (R.A.); e.gomez@ub.edu (E.G.); 2 Empa, Swiss Federal Laboratories for Materials Science and Technology, Laboratory for Mechanics of Materials and Nanostructures, Feuerwerkerstrasse 39, CH-3602 Thun, Switzerland; johann.michler@empa.ch (J.M.); laetitia.philippe@empa.ch (L.P.); 3 Institute of Nanoscience and Nanotechnology (IN2UB), University of Barcelona, E-08028 Barcelona, Spain

**Keywords:** mesoporous materials, electrodeposition, soft-templated electrodeposition, electrocatalysis, methanol oxidation

## Abstract

The use of soft templates for the electrosynthesis of mesoporous materials has shown tremendous potential in energy and environmental domains. Among all the approaches that have been featured in the literature, block copolymer-templated electrodeposition had robustness and a simple method, but it practically cannot be used for the synthesis of mesoporous materials not based on Pt or Au. Nonetheless, extending and understanding the possibilities and limitations of block copolymer-templated electrodeposition to other materials and substrates is still challenging. Herein, a critical analysis of the role of the solution’s primary electroactive components and the applied potential were performed in order to understand their influences on the mesostructure of Ni-rich Ni-Pt mesoporous films. Among all the components, tetrahydrofuran and a platinum (IV) complex were shown to be crucial for the formation of a truly 3D mesoporous network. The electrosynthesized well-ordered mesoporous Ni-rich Ni-Pt deposits exhibit excellent electrocatalytic performance for methanol oxidation in alkaline conditions, improved stability and durability after 1000 cycles, and minimal CO poisoning.

## 1. Introduction

As global warming’s dramatic effects on ecosystems escalate due to rising carbon dioxide emissions into the atmosphere, the demand for clean renewable energies has intensified research on sustainable technologies able to replace ones dependent upon exhaustible fossil fuels [1,2,3]. Among the various possibilities, fuel cells and supercapacitors are promising ecofriendly electrochemical technologies for energy conversion and storage, respectively, thanks to their tremendous potential to improve energy efficiency and reduce both carbon dioxide and nitrogen oxide emissions [4,5].

The transition to future energy systems has involved not only the development of efficient fuel cells with high power and energy density, as well as long-term stability, but also the use of virtually inexhaustible fuels (e.g., hydrogen and alcohols). Of the various types of fuel cells, direct methanol fuel cells (DMFCs) have received enormous attention, primarily due to the ease of manipulating and transporting methanol, a liquid substance that does not require special instrumentation or storage conditions. The other benefits of DMFCs include high energy density, high conversion efficiencies, negligible environmental impacts, and the ability to operate at low temperatures, the last of which significantly simplifies engineering problems [6,7,8,9].

However, using methanol in fuel cells also poses certain challenges related to unsatisfactory electrochemical performance—namely, the high anodic overpotential of methanol oxidation due to poor reaction kinetics—along with the high cost of cell components, and limited fuel cell durability [9,10]. The high cost derives principally from the use of noble metal catalysts and perfluorosulfonate polymer electrolyte membranes. On the long list of such catalysts studied to date, platinum is the most commonly used in DMFCs; however, its efficiency needs to be improved and its associated risk of carbon monoxide poisoning needs to be reduced [11,12]. To that end, nanomaterials can play a key role in developing new catalysts, whose activity intimately relates to their active surface areas (i.e., active sites) [11,12,13,14,15,16]. In general, nanocatalysts are supported on substrates with high surface area (e.g., amorphous microporous carbon powder), which afford ample surface area, sustainable porosity, high electrical conductivity, and exceptional stability. In particular, Vulcan carbon black ranks among the most widely used supports in such systems; however, its implementation requires overcoming some important limitations, including electrochemical oxidation corrosion to carbon dioxide under conditions of fuel cell operation and the resulting agglomeration, usually in the form of nanoparticles, all of which significantly decrease the system’s efficiency [17,18,19].

The development of new catalysts for DMFCs is pivotal to reducing the use of expensive Pt. As a partial solution, incorporating lower-cost transition metals to form stable bimetallic alloys—that is, Pt-M, in which “M” can be Fe, Co, Ni, or Pb, among others—has proven efficient, because it not only helps to reduce Pt consumption but also enhances the catalytic activity possible due to synergistic, electronic, and/or lattice-shrinking or straining effects. However, the chemical stability of Pt-M alloys, especially in acidic media, is generally lower than pure Pt due to the leaching of transition metals [7,12,20].

Currently, one of the most widely studied alternatives to mitigate the use of Pt and Pt-based electrocatalysts is using porous materials, especially mesoporous ones, which offer high surface-to-volume ratios, use less expensive noble materials that incorporate non-precious metals, and afford more reaction sites than their bulk counterparts, which also lowers the weight of the fuel cells [15,21,22,23]. Mesoporous materials have been synthesized using various methods, including sol-gel, direct oxidation, hydrothermal and solvothermal techniques, micelle and inverse micelle systems, chemical vapor deposition, physical vapor deposition, electrodeposition, sonochemical methods, and microwave-based techniques. All of those processes can be included in soft-templating or endotemplating methods with surfactant and block copolymers and hard-templating or exotemplating methods—that is, nanocasting. The synthesis of ordered, uniform mesopores usually requires using template or structure directors (i.e., porogen agents) in order to define the pores [20,21,22,23,24,25,26,27,28,29,30,31,32].

In that context, electrodeposition has emerged as one of the most promising technologies due to its associated advantages, especially ones stemming from its low, temporary cost and the simplicity of the experimental setup used. In particular, various approaches involving electrochemical methods that use soft and/or hard templates or electrochemical dealloying have been proposed for synthesizing complex mesoporous micro- and nano-architectured catalysts. Among all of those approaches, electrochemical dealloying, which is normally based on the selective electrochemical etching of the less noble element, has been extensively used to synthesize Pt-based mesoporous architectures. Although electrochemical dealloying offers greater control over pore formation than chemical dealloying does, the strategy affords relatively low control over the size and distribution of pores and accommodates a highly limited number of materials [22,33,34,35,36]. During the past decade, electrochemical methods using soft templates (e.g., electrodeposition using surfactants, block copolymers, supramolecular aggregates, and microemulsions) have increasingly been used due to their relatively high control over pore size distribution and pore definition, simplicity (e.g., typically a one-step procedure), low cost, potential scalability, and versatility with a wide range of materials, compositions, and architectures [22]. The simplicity of soft-template electrochemical deposition allows the synthesis of mesoporous films, nanowires, and even complex 3D architectures of metals and alloys with large surface areas due to the creation of 3D networks [22,25,26]. Not all soft-template systems have the same robustness, however, for sometimes only superficial porosity is attained.

As a partial antidote, the block copolymer-templated electrodeposition has been revealed as a reliable strategy for synthesizing Au- or Pt-based architectures that provide uniform controllable mesoporosity on metallic substrates [21,24,25,26,37,38,39]. Even so, block copolymer-templated electrodeposition offers not only excellent control of pore size and wall thickness by varying the length of copolymer fragments, but also the final composition of deposits by adjusting the parameters of electrodeposition (e.g., bath composition, temperature, and applied potential). At the same time, because copolymers with high molecular mass are not soluble in water, using organic solvents is necessary in order to dissolve the block copolymer. Regardless of the need to introduce an organic solvent, the block copolymer concentration exceeds that of critical micelles (CMC), which promotes the formation of polymeric micelles as the porogen agent for block copolymer-templated electrodeposition. That process, in turn, allows the interaction of negatively charged metal complexes with the hydrophilic part of the micelles. As a result, the codeposition of mesoporous films can be accomplished by applying an electrochemical signal [21,24,25,26,37,38,39]. Nevertheless, its use in non-Pt- or Au-based materials is highly restricted, because only Ni or Cu films can be successfully synthesized [21,24,25,26,37,38,39]. Understanding the possibilities and limitations of block copolymer-templated electrodeposition and extending the usage to other materials and other substrates is still highly required for real applications.

Herein, a systematic study, in order to clarify the role and influence of all the solution’s primary electroactive components involved in the synthesis of Ni-rich Ni-Pt well-defined 3D mesoporous networks on two typical and different substrates using block copolymer-templated electrodeposition, was performed. The results of the systematic study clarified the effect of each component of the polymeric micelle system and the influence of the electrodeposition conditions (i.e., substrate and applied potential). Tetrahydrofuran (THF), platinum (IV) ([PtCl_6_]^2−^) complex, and applied potential are crucial in mesopores definition. After clarifying the role of each component, well-ordered mesoporous Ni-rich Ni-Pt deposits were electrosynthesized and the electrocatalytic performance for the electro-oxidation of methanol in alkaline conditions was explored at room temperature. The Ni-rich Ni-Pt films are very effective for methanol electro-oxidation as a result of the highly effective superficial area, minimal CO poisoning, and enhanced stability and durability, which are promising candidates for electrocatalysis.

## 2. Materials and Methods

### 2.1. Materials

The solid—Ni(OAc)_2_∙4H_2_O (>98%), Na_2_PtCl_6_·6H_2_O (>98%), and KOH (≥85%)—and liquid compounds—ethanol (>99.8%), methanol (>99.9%), sulfuric acid (≥97.5%), and tetrahydrofuran (>99.9%)—needed to prepare the various solutions were purchased from Sigma-Aldrich (St. Louis, MO, USA). By contrast, the block copolymer polystyrene-*b*-poly (ethylene oxide) (PS-*b*-PEO) was purchased from Polymer Source Inc. (Dorval, QC, Canada). The molecular weight of each block was 63,000 for the PS and 26,000 for the PEO, respectively. All reagents were used without any prior purification and all solutions were prepared with MilliQ water from Millipore (Burlington, MA, USA).

### 2.2. Electrochemical Media

**Scheme 1:**
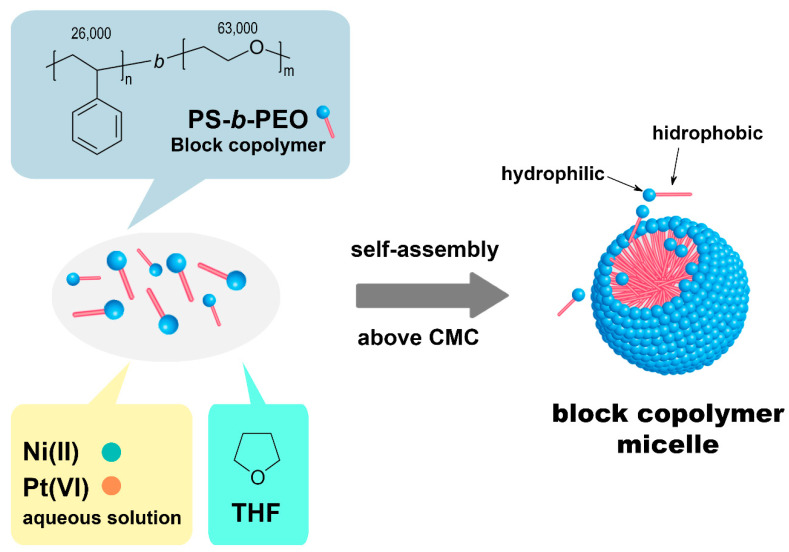
Illustration of block copolymer micelles formation.

The block copolymer-templated electrodeposition of mesoporous materials is based on the use of block copolymer micelles (Scheme 1). The polymeric micelles are formed as a result of the interaction between the hydrophilic (polar) part of the block copolymer, which corresponds to the polyethylene oxide (PEO) chains, and the water molecules, as water is the predominant solvent in the system. On the other hand, the hydrophobic (nonpolar) part of the copolymer, the polystyrene (PS) units that are not soluble in aqueous media remain inside the polymeric micelles due to their repulsive interactions with water. As nonrigid and dynamic systems, all the components can participate in the electrodeposition process. To analyze all of the individual and collective effects of each component in the electrochemical bath, the solutions summarized in Table 1 were prepared following the three-step procedure used for the preparation of the electrochemical bath used for the electrodeposition of Ni-rich Ni-Pt mesoporous films (i.e., the Ni-Pt solution in Table 1): First, 20 mg of PS-*b*-PEO was dissolved in 6 mL of THF at 50 °C. Second, once dissolved, 3 mL of ethanol and 2 mL of 80 mM Ni(OAc)_2_ + 5 mM Na_2_PtCl_6_ aqueous solution were consecutively added under magnetic stirring (450 rpm). Third, 5 mL of 0.5 M H_2_SO_4_ solution was slowly added to the solution that was maintained under magnetic stirring at 450 rpm at room temperature for 5 h.

### 2.3. Electrochemical Characterization

The electrochemical characterization of the systems was performed in a three-electrode cell with a potentiostat–galvanostat (Autolab PGSTAT30, Metrohm Autolab BV, Utrecht, Netherlands) equipped with GPES software (version 4.9). The working electrodes used were fluorine-doped tin oxide (FTO) and Si/Ti (15 nm)/Au (100 nm) referred to hereafter as Si/Ti/Au, with a geometrical area of 0.2 cm^2^ (1 × 0.2 cm for FTO and 0.5 × 0.4 cm for Si/Ti/Au). Prior to use, the working electrodes were washed with absolute ethanol, rinsed with MilliQ water, and dried with nitrogen gas. A platinum spiral cleaned with flame annealing was used as the counter-electrode. For a reference electrode, an electrode of Ag|AgCl|KCl (3M)|SO_4_^2−^ (0.5 M) was used. The potential limits were set at −1.5 and 0.4 V (vs. Ag|AgCl). All measurements were performed at 25 ± 0.1 °C under stationary conditions.

### 2.4. Electrodeposition and Characterization of Ni-Rich Ni-Pt Mesoporous Films

The Ni and Pt were codeposited using Ni-Pt solution by applying different potentials (i.e., −1.2 and −1.3 V vs. Ag|AgCl) at 25 ± 0.1 °C under stationary conditions until attaining a fixed charge density of 7.5 C cm^−2^. Because the chief objective of the study was the electrodeposition of Ni-rich Ni-Pt mesoporous films, potentials were selected, according voltammetric results, in order to ensure high Ni content. After electrodeposition, the Ni-Pt mesoporous films were washed with THF at 40 ± 0.1 °C for 30 s and rinsed with MilliQ water in order to remove deposited polymers. To study the influence of each of the system’s components in the overall deposition process, parallel electrochemical characterization was also performed using the THF, the PS-*b*-PEO, and the Ni baths (Baths 1, 2, and 3, respectively).

Deposits were morphologically, architecturally, and elementally characterized by using field-emission scanning electron microscopy (FE-SEM) with Jeol JSM 7100 F (Tokyo, Japan) and Hitachi S-4800 (Ibaraki, Japan), both of which were equipped with an energy-dispersive X-ray spectroscopy detector. All of the samples were characterized before and after being cleaned with 100 W of O_2_ plasma for 45 min. The thickness of deposits was determined by using a 3D optical surface metrology system (Leica DCM 3D, Wetzlar, Germany). The elemental composition was also examined by using X-ray fluorescence (XRF) spectrometry with a Fischerscope X-ray XDV-SDD (Fischer Instruments, Barcelona, Spain). In some samples, X-ray photoelectron spectroscopy (XPS) was also used to verify the deposition of metal. XPS data were acquired using the PHI ESCA-5500 Multitechnique system (Physical Electronics, Feldkirchen, Germany) at a base pressure of 5 × 10^−10^ mbar using monochromatic Al K-alpha radiation (i.e., Al K_α_ line of 1486.6 eV energy and 350 W) as the excitation source. The Brunauer–Emmett–Teller (BET) surface areas were measured from N_2_ adsorption–desorption isotherms at 77 K using a Micrometrics Tristar-II (Micrometrics, Ottawa, ON, Canada). The crystal phases were determined by X-ray diffraction (XRD) with a Bruker D8 Discovery diffractometer (Billerica, MA, USA) in the Bragg–Brentano configuration with Cu K_α_ radiation.

### 2.5. Electrocatalytic Performance

The electrocatalytic performance of the prepared mesoporous films for methanol electro-oxidation was evaluated in alkaline conditions (KOH 1 M). The measurements were taken in a three-electrode cell using a potentiostat–galvanostat (Autolab PGSTAT30, Metrohm Autolab BV, Utrecht, Netherlands) equipped with Nova software (version 2.1). Mesoporous films, Pt spirals, and Ag|AgCl|KCl (3 M)|SO_4_^2−^ (0.5 M) were used as working, counter, and reference electrodes, respectively. The working electrode was previously subjected to 40 cyclic voltammograms between −1.5 and 0.4 V at 10 mV s^−1^ for conditioning in a KOH solution of 1 M at 25 ± 0.1 °C. The methanol oxidation reaction (MOR) was studied in solutions of 1 M methanol in 1 M KOH solution using the same potential window and a scan rate of 50 mV s^−1^. The stability and durability of the prepared mesoporous films were examined by measuring the catalytic performance after 1000 cycles in 1 M methanol + 1 M of KOH solution using the same potential window and a scan rate of 100 mV s^−1^.

## 3. Results

A systematic study of the various components of the system was performed in order to identify the limitations and robustness of using block copolymer-templated electrodeposition to electrosynthesize mesoporous materials. The effects of two typical electrodes (i.e., FTO-coated glass and Si/Ti/Au electrodes) ideal for use in a wide range of applications, including optoelectronics, thin-film photovoltaics, energy-saving windows, and catalysis, were also examined.

### 3.1. Electrochemical Characterization

**Figure 1:**
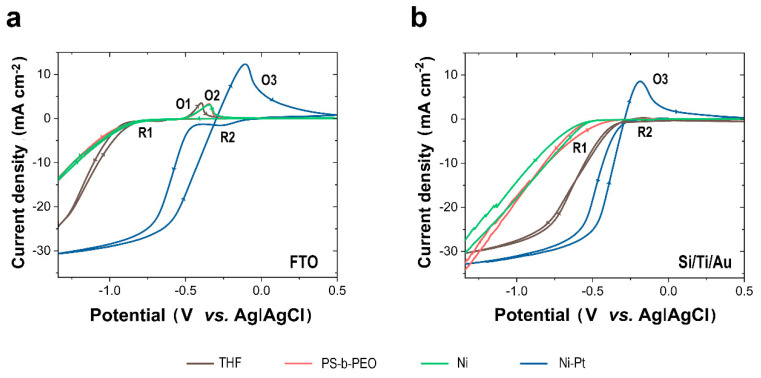
Cyclic voltammograms of the four electrochemical baths (i.e., THF (black), PS-*b*-PEO (red), Ni (green), and Ni-Pt (blue)) on (**a**) FTO-coated glass and (**b**) Si/Ti/Au at 50 mV s^−1^ and 25 °C.

To study the electrochemical behavior of block copolymer-templated electrodeposition, voltammetric studies on the FTO and Si/Ti/Au substrates were performed in four different electrochemical baths (Table 1), all at a controlled temperature of 25 °C and in stationary conditions. As shown in Figure 1a, using FTO, similar electrochemical behavior was observed in the THF (i.e., Bath 1), PS-*b*-PEO (i.e., Bath 2), and Ni (i.e., Bath 3) solutions, all of which demonstrated a clear reduction current (R1) on the cathodic scan at approximately −0.8 V (vs. Ag|AgCl). Although analogous general behavior was observed with the Si/Ti/Au electrodes (Figure 1b), the reduction peaks in the PS-*b*-PEO (Bath 2) and Ni solutions (Bath 3) began at slightly more negative potentials of around −0.65 V (vs. Ag|AgCl) than with the THF solution (Bath 1), which began peaking at approximately −0.5 V (vs. Ag|AgCl). As easily observable on the slope of the reduction peak, the reduction in the THF solution (Bath 1) occurred at a higher rate, especially in the case of FTO. That outcome suggests that THF participated in the reduction process as the formation of polymeric micelles (i.e., PS-*b*-PEO and Ni solutions), which partially blocked THF’s interaction with the electrode’s surface and significantly reduced the kinetics of electroreduction. In a previous study, when Ni solution was studied on Si/Ti/Au, the reduction peak at approximately −0.8 V (vs. Ag|AgCl) was assigned to the simultaneous Ni(II) reduction to metallic Ni and the hydrogen evolution reaction [37]. Note that the cyclic voltammogram profiles in the presence and in the absence of Ni(II) in both PS-*b*-PEO and Ni solutions were identical.

By contrast, the anodic scan on FTO revealed a clear oxidation peak (O1) at approximately −0.55 V (vs. Ag|AgCl) in the THF solution and another (O2) at approximately −0.60 V (vs. Ag|AgCl) in the PS-*b*-PEO and Ni solutions. All of those oxidation peaks rose significantly in the oxidation scan after the potential was maintained at −1.3 V (vs. Ag|AgCl) during 60 s in the negative scan under the same conditions. A thin layer could be observed on the FTO electrodes after 60 s of deposition at −1.3 V. However, no oxidation peaks—that is, neither O1 nor O2—were recorded when Si/Ti/Au was used as a working electrode. It is well known that FTO catalyzes the electrodeposition of various polymers such as polypyrrole, polyaniline, poly(3,4-ethylenedioxythiophene), and polybithiophene [39,40,41]. Consequently, the reduction peak observed with the THF (Bath 1), PS-*b*-PEO (Bath 2), and Ni (Bath 3) solutions can be assumed to indicate the simultaneous deposition of THF, PS-*b*-PEO, and/or Ni, depending on the system, as well as the hydrogen evolution reaction. Therefore, the oxidation peak O1 could be assigned to the polytetrahydrofuran’s electro-oxidation, whereas O2 could be ascribed to the partial oxidation of the deposited block copolymer. The codeposition of polymers could benefit the formation of decorated or even mesoporous architectures. Previous studies have demonstrated the codeposition of metals and spherical polymeric micelles present in the electrolyte, which prompted the formation of mesoporous metallic structures following the dissolution of the polymers [42,43,44]. After all, the surface charge of polymeric micelles is a key factor for attraction to the working electrode’s surface, as well as for the selection of the system’s constituents. In that way, well-defined metallic mesoporous materials are deposited, because the global surface charge of the polymeric micelles should be positive to be electrostatically attracted to the negatively polarized surface of the working electrode.

However, different voltammetric behavior was observed in the presence of the [PtCl_6_]^2−^ complex (Bath 4), as evidenced by the advance of the reduction peak’s (R_2_) onset to approximately −0.5 V (vs. Ag|AgCl) on FTO and −0.4 V (vs. Ag|AgCl) on Si/Ti/Au, under the influence of the interaction of [PtCl_6_]^2−^ anions in the hydrophilic part of the polymeric micelles. The intensity of the subsequent reduction peak also increased due to the promotion of the hydrogen evolution reaction on the previously deposited Ni-Pt. In the anodic scan, a clear oxidation peak (O3) at approximately −0.30 V (vs. Ag|AgCl) was observed, which was ascribed to the Ni-Pt’s oxidation. Such behavior suggests that the interaction between the Pt complex and the hydrophilic part of the polymeric micelles promoted the codeposition of Pt and the copolymer; the freshly deposited metal unambiguously promoted the codeposition of Ni, and enhanced the hydrogen evolution reaction.

### 3.2. Analysis of the Potential Interference of Organic Component and Block Copolymer Micelles on the Deposition of Ni-Rich Ni-Pt Mesoporous Films

Based on the cyclic voltammetric study, Ni-rich Ni-Pt mesoporous films were potentiostatically electrodeposited at −1.3 and −1.2 V (vs. Ag|AgCl) in the Ni-Pt (Bath 4) solution under stationary conditions until the same deposition charge density (7.5 C cm^−^^2^) was attained. To analyze the potential interference of each component of the block copolymer micellar system, electrodeposition in the same conditions at −1.3 V (vs. Ag|AgCl) was also performed using the THF (Bath 1) and PS-*b*-PEO (Bath 2) solutions. After electrodeposition, all of the samples were cleaned with pure THF at 40 °C in order to remove the remaining block copolymer. Next, the samples were subjected to 100 W of O_2_ plasma for 45 min to remove the deposited organic matter. Among the results, the first difference observed was that, to the naked eye, the samples prepared on Si/Ti/Au did not reveal the presence of any deposit, whereas a gray, metal-like coating was observed on FTO in all cases.

**Figure 2:**
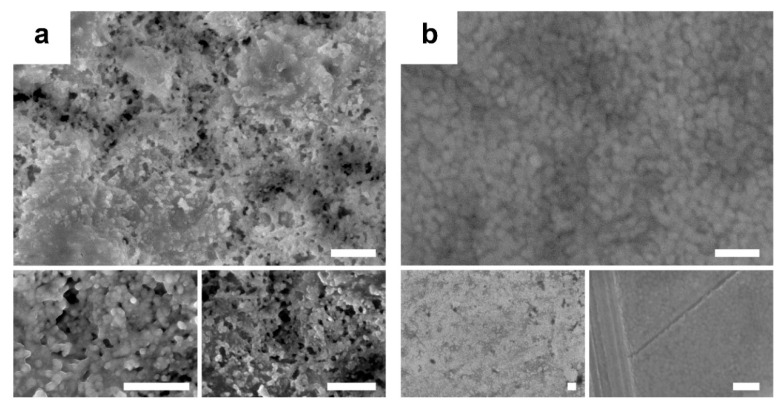
FE-SEM micrographs at different magnifications of the deposits obtained using the THF (Bath 1) solution on (**a**) FTO-coated glass and (**b**) Si/Ti/Au substrates at −1.3 V (vs. Ag|AgCl) after circulating 7.5 C cm^−2^. Scale bar: 300 nm.

**Figure 3:**
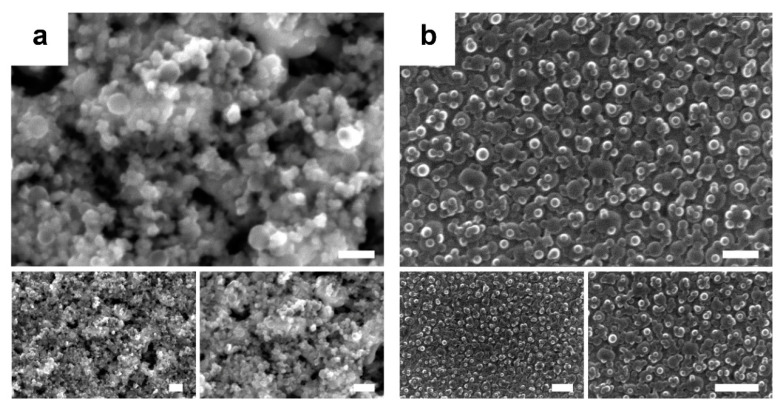
FE-SEM micrographs at different magnifications of the deposits obtained using PS-*b*-PEO (Bath 2) solution on (**a**) FTO-coated glass and (**b**) Si/Ti/Au substrates at −1.3 V (vs. Ag|AgCl) after circulating 7.5 C cm^−2^. Scale bar: 300 nm.

From the THF solution, a homogeneous tridimensional mesoporous architecture (Figure 2a) was also obtained on the FTO-coated glass, whereas no deposit was observed on Si/Ti/Au (Figure 2b). As the results of the preliminary electrochemical study suggested would occur, the electrodeposition of the polytetrahydrofuran was catalyzed by FTO-coated glass only. By contrast, the electrodeposits obtained using the PS-*b*-PEO solution revealed the formation of a fluffy film on FTO (Figure 3a) and compact films with spherical grains on Si/Ti/Au (Figure 3b), both due to the electropolymerization of the block copolymer. The morphological analysis of those samples was performed prior to cleaning the deposit with THF and O_2_ plasma in order to check the true morphology of the deposits. Both polymeric films were greatly affected and entirely removed when subjected to O_2_ plasma for a prolonged period (>120 min).

The electropolymerization of both THF and block copolymer must always be considered when a block copolymer-templated electrodeposition is used for the synthesis of mesoporous materials. As block copolymer micelles are dynamic systems, it is expected that both THF and the block copolymer also interacted with the working electrode surface during electrodeposition, and consequently, codeposited simultaneously with other electroactive species like Ni(II) or Pt(VI). This process can be beneficial for the creation of pores, if polymers are etched or dissolved, but can also reduce mesopores definition or even film integrity.

### 3.3. Electrodeposition and Characterization of Ni-Rich Ni-Pt Mesoporous Films

**Figure 4:**
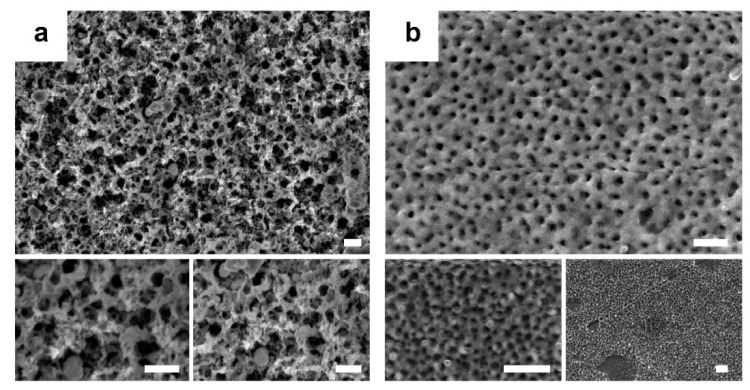
FE-SEM micrographs at different magnifications of the deposits obtained using Ni (Bath 3) solution on (**a**) FTO-coated glass and (**b**) Si/Ti/Au substrates at −1.3 V (vs. Ag|AgCl) after circulating 7.5 C cm^−2^. Scale bar: 300 nm.

With the aim to reduce the use of expensive noble metals in electrodeposition, as well as to analyze the potential of block copolymer-templated electrodeposition for materials not based on Pt or Au, electrodeposition was performed in the Ni (Bath 3) solution. The deposits obtained from that solution (Figure 4a) on FTO presented a 3D sponge network morphology, with pores ranging from approximately 80 to 200 nm, which highlights the extremely relevant role of the ionic salt used in the deposition.

When the Ni(OAc)_2_ precursor was used in acidic conditions, the electrostatic repulsion of positively charged Ni(II) ions with the positively charged polymeric micelles inhibited their interaction. However, minimal adsorption of positively charged Ni(II) ions via hydrogen bonding was expected [37]. The primary Ni(II) species in the aqueous solution in those conditions was Ni^2+^ ions. Because the non-nickel functionalized positively charged polymeric micelles were attracted to the working electrode, the definition of mesopores was poor, and the effect of THF’s deposition was more important. After all, polymeric micelles are dynamic systems and THF is not entirely physically retained in the inner part of the polymeric micelles. The elemental analysis of those deposits confirmed the global codeposition of Ni; however, Ni was not locally detected even in the XPS analysis of various regions of the deposits (Figure S1, Supplementary Material). Because the deposit was partially removed when subjected to an O_2_ plasma treatment (Figure S2, Supplementary Material), Ni-decorated microporous films could be easily obtained using block copolymer-templated electrodeposition in those conditions.

The electrodeposition on Si/Ti/Au, in which the electropolymerization of THF was not catalyzed, facilitated the codeposition of Ni and the block copolymer, thereby prompting the formation of mesoporous Ni films (Figure 4b). However, the efficiency of electrodeposition was extremely low, and deposits were heterogeneously distributed in the corners of the working electrode. In those samples, three regions could be easily distinguished. The first was the Si/Ti/Au surface without any deposit, which encompassed most of the surface. The second region contained compact films of copolymer (Figure S3, Supplementary Material) that were identical to those obtained with the PS-*b*-PEO solution (Figure 3b), which can be attributed to the deposition of polymeric micelles that did not contain adsorbed Ni^2+^ ions. The last region consisted of mesoporous Ni films at the corners of the working electrode (Figure 4b). Due to the heterogeneity of the deposits, the measured average thickness ranging from <100 to >600 nm clearly observed for the samples has not been reported. Therefore, using block copolymer-templated electrodeposition to prepare continuous mesoporous Ni needs to be improved, particularly using other Ni precursors that can electrostatically interact with the hydrophilic part of polymeric micelles, whose surface charge was positive, which promoted access to the electrode and overcame the intrinsically inert character of Ni during electrodeposition.

**Figure 5:**
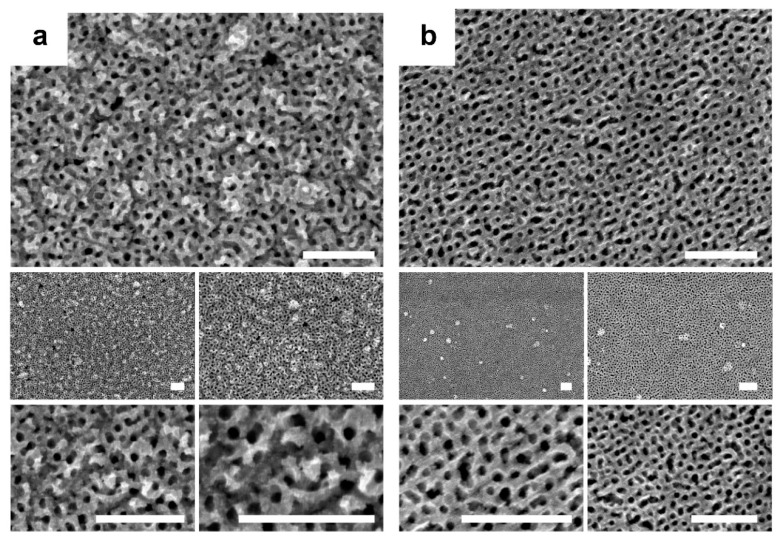
FE-SEM micrographs at different magnifications of the deposits obtained using Ni-Pt (Bath 4) solution on (**a**) FTO-coated glass and (**b**) Si/Ti/Au substrates at −1.3 V (vs. Ag|AgCl) after circulating 7.5 C cm^−2^. Scale bar: 300 nm.

Last, using the Ni-Pt (Bath 4) solution, in which the negatively charged platinum (IV) chloro complexes were the primary platinum species (i.e., in the working conditions), mesoporous films of Ni-rich Ni-Pt were electrosynthesized. The coexisting platinum (IV) chloro complexes in aqueous solution interacted electrostatically with the hydrophilic part of the polymeric micelles, thereby rendering the global surface charge of Pt-functionalized micelles positive, even in the presence of Ni(II), and thus, favoring the attraction of polymeric micelles on the negatively polarized surface of the working electrode. In that dynamic, the polymeric micelles functioned as the porogen agent used in block copolymer-templated electrodeposition. As shown in Figure 5a,b, 3D mesoporous films, with pore diameters ranging from approximately 30 to 35 nm, were electrodeposited over the entire surface of FTO and Si/Ti/Au electrodes. Consequently, selecting an appropriate metallic precursor is pivotal to guarantee definition of mesopores in block copolymer-templated electrodeposition.

By changing the electrodeposition potential, the Ni/Pt ratio could be modified in the synthesized mesoporous films. When most negative deposition potentials were applied, the Ni content in the films was higher. By contrast, a decrease in the porosity of the films was also observed when Ni content increased. This dynamic confirms the relevance of the [PtCl_6_]^2−^ complex to the system as the species that interacts with the hydrophilic part of the polymeric micelles and, in that role, prompts the deposition of well-defined, uniformly distributed Ni-rich Ni-Pt mesoporous films. Indeed, the described interaction is the determining factor in the production of mesoporous films, because compact deposits would be obtained otherwise. Therefore, a compromise between Ni content and the integrity of mesopores was required to obtain films exhibiting a mesoporous structure with the most Ni content. In those conditions, in terms of Ni content and the definition of porosity, the most adequate conditions occurred with potentiostatic electrodeposition at −1.2 (Figure S4, Supplementary Material) and −1.3 V (Figure 5), both versus Ag|AgCl, in which the mesopores were uniformly distributed over the entire surface without any visible voids or cracks. After the total removal of organic matter by O_2_ plasma treatment, the composition of the Ni-rich Ni-Pt mesoporous films (Table 2) was subjected to EDX and XRF analysis. According to the literature, Ni content can be enhanced by increasing the temperature during electrodeposition and, in turn, accelerating the transport of Ni(II) ions from the electrolyte to the working electrode. Based on such evidence, the reduction in Ni^2+^/Ni can hardly occur at temperatures below 40 °C. However, in the experiment described here, the Ni-rich Ni-Pt mesoporous films were successfully electrodeposited at 25 °C.

**Figure 6:**
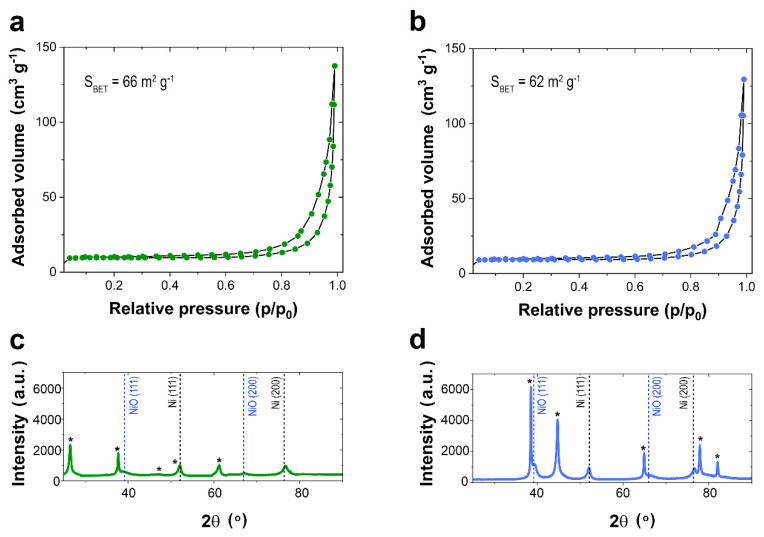
(**a**,**b**) Nitrogen adsorption and desorption isotherms and (**c**,**d**) XRD patterns of Ni-rich Ni-Pt mesoporous films prepared at −1.3 V (vs. Ag|AgCl) on (**a**) FTO and (**b**) Si/Ti/Au substrates. The diffraction peaks that correspond to the FTO and Si/Ti/Au substrates, respectively, are indicated by *.

The Ni-rich Ni-Pt mesoporous films prepared at −1.3 V (vs. Ag|AgCl) were subjected to nitrogen gas adsorption and desorption in order to determine the BET surface areas. As shown in Figure 6a,b, a gradual increase in adsorbed nitrogen up to relative pressure of approximately 0.75 was observed, followed by a hysteresis loop between relative pressure values of 0.75 to 0.995, which suggests the mesoporous nature of both films. The Ni-rich Ni-Pt mesoporous films prepared on FTO exhibited a BET surface area of 66 m^2^ g^−1^ and an average pore diameter of 26 nm, whereas the ones prepared on Si/Ti/Au exhibited a BET surface area of 62 m^2^ g^−1^ and an average pore diameter of 25 nm. The average pore diameters agreed well with results obtained earlier from FE-SEM analysis. Therefore, the prepared Ni-rich Ni-Pt mesoporous films should be excellent candidates with high-performance electrocatalytic activity for MOR due to the enhanced surface area.

The XRD patterns (Figure 6c,d) show the (111) and (200) reflections for Ni face-centered cubic (*fcc*) crystal phase, along with the reflections of the FTO (Figure 6c) and Au (Figure 6d) seed layers. However, the (111) and (200) reflections for NiO *fcc* crystal phase were also detected, which was expected due to the superficial oxidation possible during the oxygen plasma treatment that removes the codeposited organic matter. The formation of NiO can also benefit MOR in alkaline conditions.

Consequently, well-defined bimetallic Ni-rich Ni-Pt alloyed mesoporous films, easily synthesized using block copolymer-templated electrodeposition, on FTO-coated glass and Si/Ti/Au electrodes, are expected to possess excellent electrocatalytic performance for MOR due to the large surface area and the synergetic effects of the architecture combined with the chemical and electronic properties.

### 3.4. Electrocatalytic Performance

**Figure 7:**
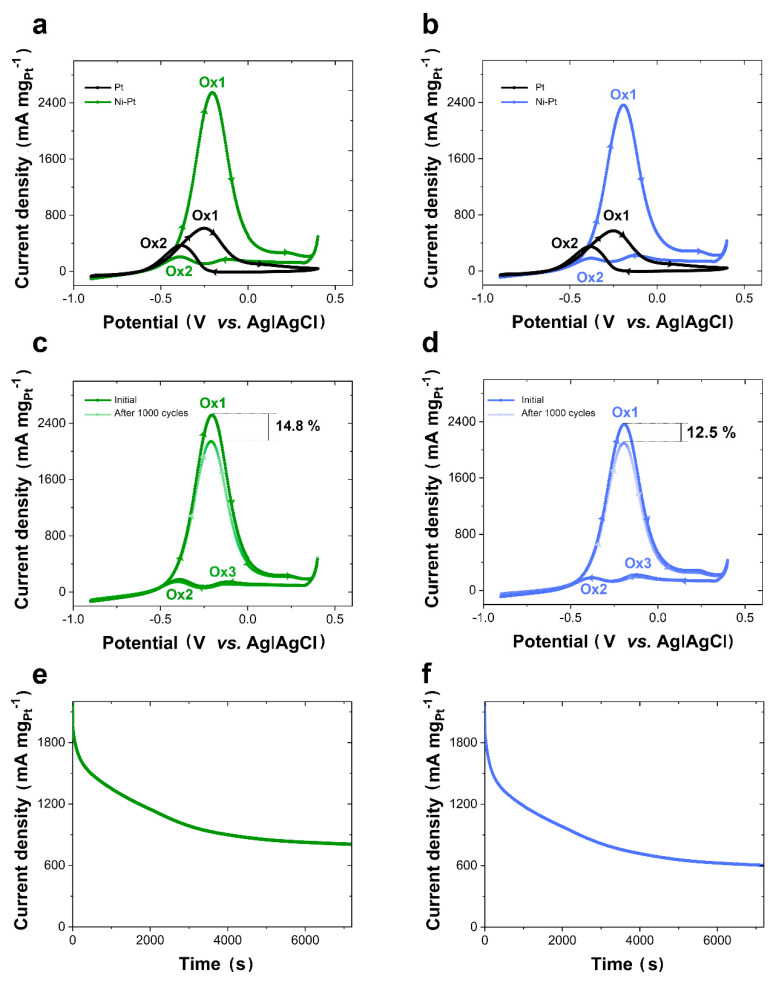
Cyclic voltammograms of methanol electro-oxidation in a solution containing 1 M of KOH and 1 M of methanol at a scan rate of 50 mV s^−1^ for Pt and Ni-rich Ni-Pt mesoporous films electrodeposited on (**a**) FTO and (**b**) Si/Ti/Au substrates. Cyclic voltammograms of methanol electro-oxidation in a solution containing 1 M of KOH and 1 M of methanol at a scan rate of 50 mV s^−1^ before and after 1000 cycles for Ni-rich Ni-Pt mesoporous films deposited on (**c**) FTO and (**d**) Si/Ti/Au substrates, along with chronoamperometric curves recorded at −0.15 V versus Ag|AgCl for 7200 s for Ni-rich Ni-Pt mesoporous films deposited on (**e**) FTO and (**f**) Si/Ti/Au substrates. T = 25 °C.

The electrocatalytic properties of Ni-rich Ni-Pt mesoporous films for the methanol oxidation reaction (MOR) on FTO-coated glass and Si/Ti/Au substrates were compared to pure Pt mesoporous films at 1 M of KOH containing 1 M of methanol using cyclic voltammetry (CV), as shown in Figure 7. Once the currents were normalized using the Pt mass of electrodeposits, the CV profiles revealed similar methanol oxidation peaks in the forward scan (Ox1) for FTO and Si/Ti/Au electrodes. Such peaks can be ascribed to the electro-oxidation of freshly chemisorbed methanol, whereas a significantly smaller oxidation peak in the backward scan (Ox2) can be ascribed to the removal and oxidation of residually adsorbed carbonaceous intermediates produced during the forward scan [45,46,47]. As expected, the mass activity (i.e., current normalized by Pt mass) and the surface activity (i.e., current normalized by BET surface area) of Ni-rich Ni-Pt mesoporous films were significantly (i.e., approximately 5 times) and slightly (i.e., approximately 1.2 times) higher, respectively, than the activities observed for Pt mesoporous films (i.e., independently on the substrate) due to the significantly lower amount of Pt in Ni-rich Ni-Pt mesoporous films for a virtually identical specific surface area. Both mass and surface activities were slightly higher on the FTO than on the Si/Ti/Au substrates, as consistent with their slightly higher BET surface areas. The MOR activity of Ni-rich Ni-Pt mesoporous films was comparable to, if not higher than, what has been reported in the literature for MORs in alkaline conditions with state of the art Pt-based catalysts (Table 3) [48,49,50,51,52,53,54,55,56]. The negative shift in the onset potential (*E_onset_*, Table 3) and peak potential (*E_p_*, Table 3) for both the FTO-coated glass and the Si/Ti/Au Ni-rich Ni-Pt mesoporous films indicated significant improvement in the kinetics of methanol electro-oxidation compared with that of pure Pt structures under the same experimental conditions, possibly due to the synergistic effect of mesoporous morphology and chemical composition. In addition, a third oxidation voltammetric peak (Ox3) was observed that could be attributed to the superficial oxidation of Ni-Pt mesoporous films, with a peak that was also detected in the absence of methanol, from the solution (i.e., 1 M of KOH). Ni-rich Ni-Pt mesoporous films can oxidize methanol to carbon dioxide more efficiently than pure Pt or other electrocatalysts due to the former’s higher tolerance to the intermediate carbonaceous species (e.g., CO_ads_ and CHO_ads_) accumulated on the surface of the electrodes. A major limitation of Pt electrocatalysts in MORs, however, is the low tolerance of Pt to CO_ads_ poisoning, which can be easily evaluated by using the ratio of the forward anodic peak’s current density (*j_f_*) to that of the backward anodic peak (*j_b_*). As shown in Table 3, higher *j_f_*/*j_b_* ratios indicate a higher tolerance to poisoning. In the experiment, the tolerance of the Ni-rich Ni-Pt mesoporous films was significantly higher than the state of the art Pt-based catalysts [48,49,50,51,52,53,54,55,56].

The long-term stability and durability of electrocatalysts are important parameters in determining whether Ni-rich Ni-Pt mesoporous films are promising applicable electrocatalysts for fuel cells. The durability of electrocatalysts was, therefore, initially evaluated with cyclic voltammetry (i.e., 1000 cycles using the same potential window and a scan rate of 100 mV s^−1^) and chronoamperometry (i.e., at −0.15 V vs. Ag|AgCl for 7200 s) in a solution containing 1 M of KOH and 1 M of methanol. As shown Figure 7c,d, the scanning peak currents before and after 1000 cycles exhibited an attenuation of 14.8% and 12.5% for Ni-rich Ni-Pt mesoporous films on FTO-coated glass and Si/Ti/Au substrates, respectively. Those values indicate an acceptable durability compared with the state of the art Pt-based electrocatalysts for methanol electro-oxidation in both acidic and alkaline conditions. The durability of Ni-rich Ni-Pt mesoporous films was additionally confirmed by chronoamperometry. As shown Figure 7e,f, the high steady-state current densities achieved after the initial decrease due to the generation of reactive intermediate species, which aligned with those obtained from CV, also demonstrated excellent catalytic performance compared with the state of the art Pt-based electrocatalysts. The stability and durability of the catalyst were also morphologically and structurally confirmed by using FE-SEM observation and XRD analysis (Figure S5, Supplementary Material). After the 1000-cycle test experiments, no effects were observed on either the morphology or the structure of the deposits independent of the substrate used.

## 4. Conclusions

Bimetallic Ni-rich Ni-Pt mesoporous films, which demonstrate highly accessible active surface sites, were synthetized via simple block copolymer-templated electrochemical deposition and tested as efficient electrocatalysts for methanol electro-oxidation in alkaline conditions. The possibilities and potential limitations of block copolymer-templated electrodeposition of metallic mesoporous films on two different typical working electrodes (i.e., FTO and Si/Ti/Au) were clarified by analyzing the role of each of the solution’s component and the applied potential on the pore definition. The experiments revealed that both the THF (when used as it is not necessary for all of the block copolymers) and the block copolymer electropolymerization can be significantly promoted depending on the nature of the substrate; this can be beneficial or detrimental if it is removed, depending on the deposition efficiency, for the formation of a well-defined mesoporous 3D network. Among other findings, the nature of the metallic precursor has been proven to be crucial for allowing the interaction of the hydrophilic micellar portion and the metal complex. The global charge density of micelles–metal complex should be positive to promote their electrostatic attraction to the working electrode surface. The mesoporous metallic deposition was undertaken with the initial deposition of the copolymer and platinum, the presence of which favors the consequent codeposition of nickel. The process was also accompanied by a simultaneous hydrogen evolution, related to the necessarily high proton concentration and promoted by the metallic Ni-rich Ni-Pt material. By varying the applied potential, Ni-Pt deposits with a Ni content of 65–75% and a mesoporous structure with a pore size between 20 and 35 nm were attained. The resulting mesoporous films exhibited an exceptionally effective surface area of 65 m^2^ g^−1^ on average, which presents conditions that are favorable for reactants to have maximum access to active sites, thereby highlighting them as potential candidates for electrocatalysis. Lastly, the electrochemical oxidation of methanol in an alkaline medium was analyzed in order to test the electrocatalytical performance of the aforementioned mesoporous films. The voltammetric characterization of the process revealed that the oxidation of methanol could occur over a wide range of potentials without detecting the typical inhibitory effect of the oxidation reaction intermediates, which is typically the most deleterious effect that occurs on pure platinum structures. Potentiostatic experiments conducted with the more positive potentials confirmed the stationary current value recorded in long-term experiments, which demonstrates the promising stability and durability of the prepared materials for MOR.

## Figures and Tables

**Table 1:** Electrochemical baths and bath composition.

Bath	Solution	Bath Composition
1	THF solution	6 mL of THF + 3 mL of ethanol + 2 mL H_2_O + 5 mL of 0.5 M H_2_SO_4_
2	PS-*b*-PEO solution	6 mL of THF containing 20 mg of PS-*b*-PEO + 3 mL of ethanol + 2 mL H_2_O + 5 mL of 0.5 M H_2_SO_4_
3	Ni solution	6 mL of THF containing 20 mg of PS-*b*-PEO + 3 mL of ethanol + 2 mL of 80 mM Ni(OAc)_2_ aqueous solution + 5 mL of 0.5 M H_2_SO_4_
4	Ni-Pt solution	6 mL of THF containing 20 mg of PS-*b*-PEO + 3 mL of ethanol + 2 mL of 80 mM Ni(OAc)_2_ + 5 mM Na_2_PtCl_6_ aqueous solution + 5 mL of 0.5 M H_2_SO_4_

**Table 2:** Deposition potential, average thickness, elemental composition, pore diameter, and BET surface area of the Ni-rich Ni-Pt mesoporous films.

Deposition Potential (V vs. Ag|AgCl)	Thickness (nm)		Elemental Composition (at. %)		Pore Diameter (nm)		BET Surface Area (m^2^g^−1^)	
	FTO	Si/Ti/Au	FTO	Si/Ti/Au	FTO	Si/Ti/Au	FTO	Si/Ti/Au
−1.2	~540	~490	66 ± 2% of Ni	64 ± 1% of Ni	20–30	20–30	-	-
−1.3	~520	~480	74 ± 1% of Ni	71 ± 2% of Ni	20–35	25–35	66	62

**Table 3:** Catalytic performance of Pt-based electrocatalysts for methanol electro-oxidation in a solution containing 1 M of KOH and 1 M of methanol at a scan rate of 50 mV s^−1^.

System	*E_onset_* (V)	*Ep* (V)	*j*_f_ (mA cm^−2^)	*j*_f_ (mA mg_Pt_^−1^)	*j*_f_/*j*_b_	References
Pt on FTO-coated glass	−0.588	−0.251	8.5	664	1.7	This work
Pt on Si/Ti/Au	−0.594	−0.248	7.4	573	1.6	This work
Ni-Pt on FTO-coated glass	−0.613	−0.207	10.1	2546	14.9	This work
Ni-Pt on Si/Ti/Au	−0.501	−0.192	9.4	*2364*	16.8	This work
Pt-Co NF	-	-	-	4280	-	[48]
Pt-BiOI/MoS_2_	−0.582	−0.196	-	1007	1.3	[49]
Pt/g-C_3_N_4_/MoS_2_	−0.646	−0.194	-	1618	2.8	[50]
Pt-2D Fe_2_O_3_	−0.412	−0.184	-	343	2.5	[51]
Pt-Ag/G	−0.462	−0.069	-	5628	5.6	[52]
Pt-doped Ni(OH)_2_	−0.581	0.058	-	1356	5.6	[53]
Pt-CoOOH-CDs/C	−0.578	−0.205	-	856	4.52	[54]

## Supplementary Materials

The following are available online at https://www.mdpi.com/2079-4991/10/8/1435/s1, Figure S1: XPS spectra of deposits obtained using Ni (Bath 3) solution on FTO-coated glass at −1.3 V (vs. Ag|AgCl) after circulating 7.5 C cm^−2^; Figure S2: FE-SEM micrographs of the deposits obtained using Ni (Bath 3) solution on FTO-coated glass at −1.3 V (vs. Ag|AgCl) after circulating 7.5 C cm^−2^ and being subjected to a 45 min O_2_ plasma treatment; Figure S3: FE-SEM micrographs of the deposits obtained using Ni (Bath 3) solution Si/Ti/Au substrates at −1.3 V (vs. Ag|AgCl) after circulating 7.5 C cm^−2^; and Figure S4: FE-SEM micrographs of the deposits obtained using Ni-Pt (Bath 4) solution on (a) FTO-coated glass and (b) Si/Ti/Au substrates at −1.2 V (vs. Ag|AgCl) after circulating 7.5 C cm^−2^. Figure S5: FE-SEM micrographs and XRD patterns of the deposits obtained using Ni-Pt (Bath 4) solution on FTO-coated glass and Si/Ti/Au substrates at −1.3 V (vs. Ag|AgCl) after 1000 cycling experiments.
