# Supplementary material for: Electrodeposition of Mesoporous Ni-Rich Ni-Pt Films for Highly Efficient Methanol Oxidation
Source: Nanomaterials (Basel). 2020 Jul 23;10(8):1435. doi: 10.3390/nano10081435 (PMC7466700; doi:10.3390/nano10081435)
Supplement: Supplementary file 1 [file nanomaterials-10-01435-s001.pdf]

nanomaterials

MDPI

Supplementary Material

Electrodeposition of Mesoporous Ni-Rich Ni-Pt Films for Highly Efficient Methanol Oxidation

Raül Artal [1,2,†], Albert Serrà [2,*,†], Johann Michler [2], Laëtitia Philippe [2], and Elvira Gómez [1,3]

1. Thin Films and Nanostructures Electrodeposition Group (GE-CPN), Departament of Ciència de Materials i Química Física, University of Barcelona, Martí i Franquès 1, E-08028 Barcelona, Catalonia, Spain; rartal2@gmail.com (R.A.), e.gomez@ub.edu (E.G.)
2. Empa, Swiss Federal Laboratories for Materials Science and Technology, Laboratory for Mechanics of Materials and Nanostructures, Feuerwerkerstrasse 39, CH-3602 Thun, Switzerland; johann.michler@empa.ch (J.M.); laetitia.philippe@empa.ch (L.P.)
3. Institute of Nanoscience and Nanotechnology (IN2UB), University of Barcelona, E-08028 Barcelona, Catalonia, Spain

* Correspondence: albert.serraramos@empa.ch

† These authors contributed equally.

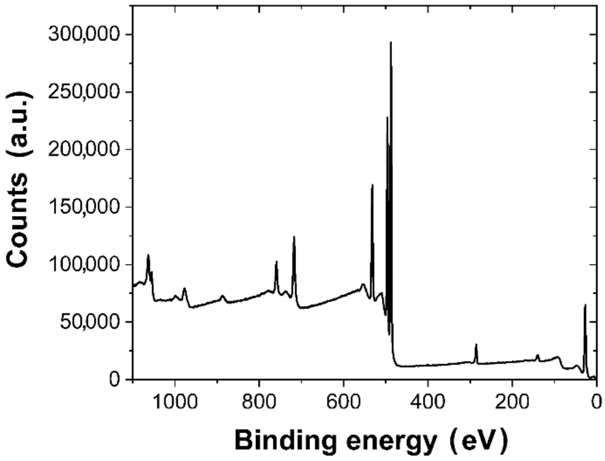

Figure S1. XPS survey spectra of deposits obtained using Ni (bath 3) solution on FTO-coated glass at −1.3 V (vs. Ag|AgCl) after circulating 7.5 C cm^{-2}.

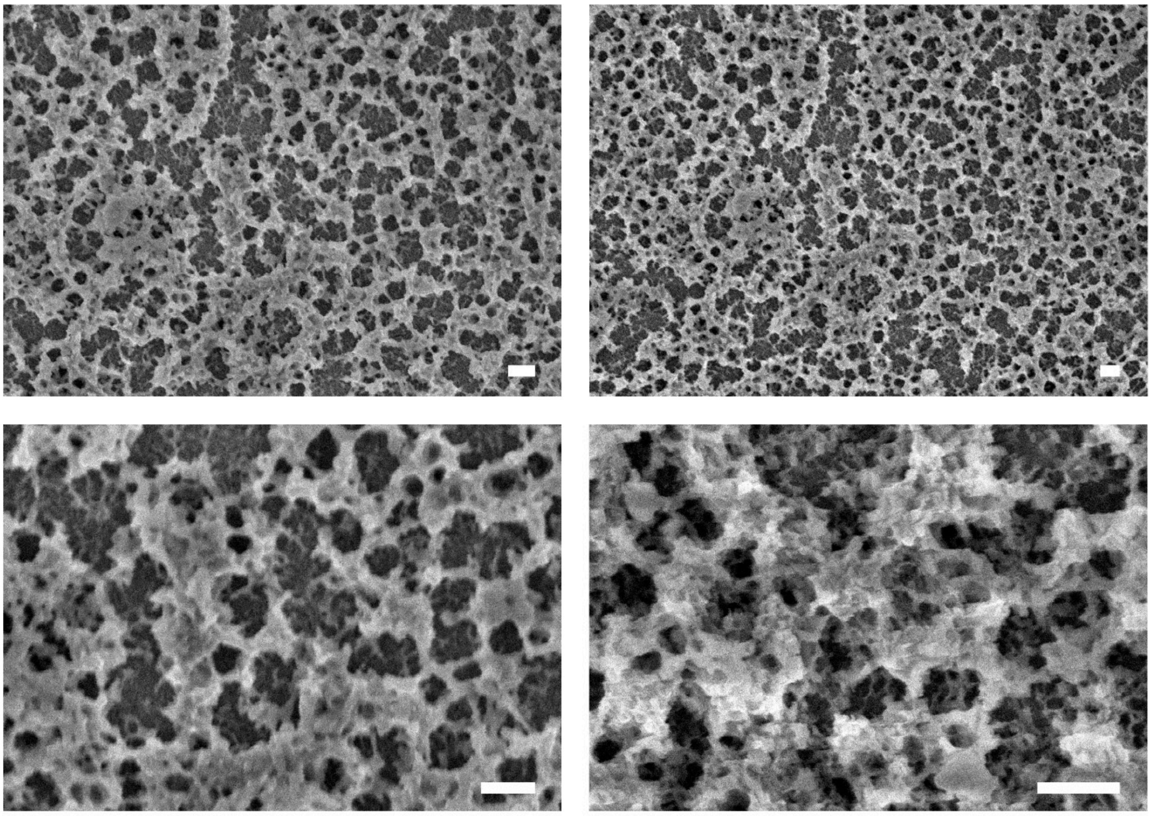

Figure S2. FE-SEM micrographs at different magnifications of the deposits obtained using Ni (bath 3) solution on FTO-coated glass at −1.3 V (vs. Ag|AgCl) after circulating 7.5 C cm^{-2} and being subjected to a 45 min O_2 plasma treatment. Scale bar: 300 nm.

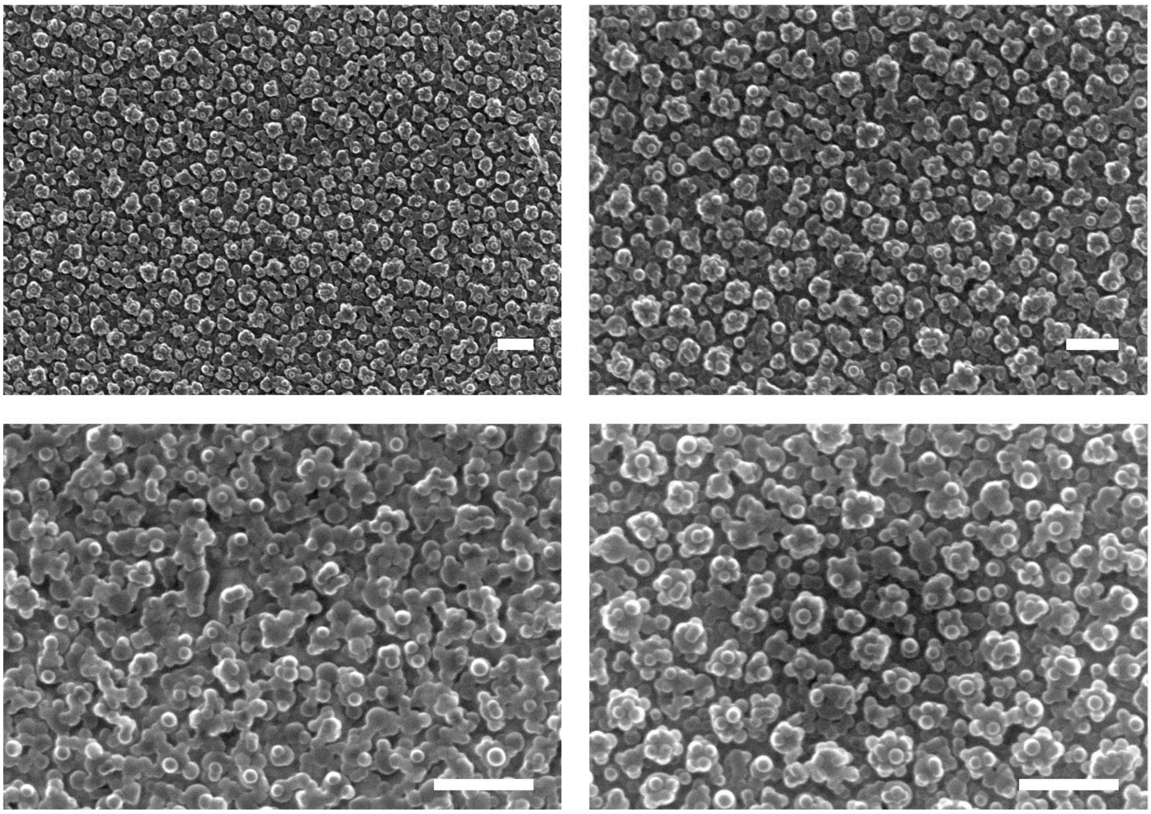

Figure S3. FE-SEM micrographs at different magnifications of the deposits obtained using Ni (bath 3) solution Si/Ti/Au substrates at −1.3 V (vs. Ag| AgCl) after circulating 7.5 C cm^{-2}. Scale bar: 300 nm.

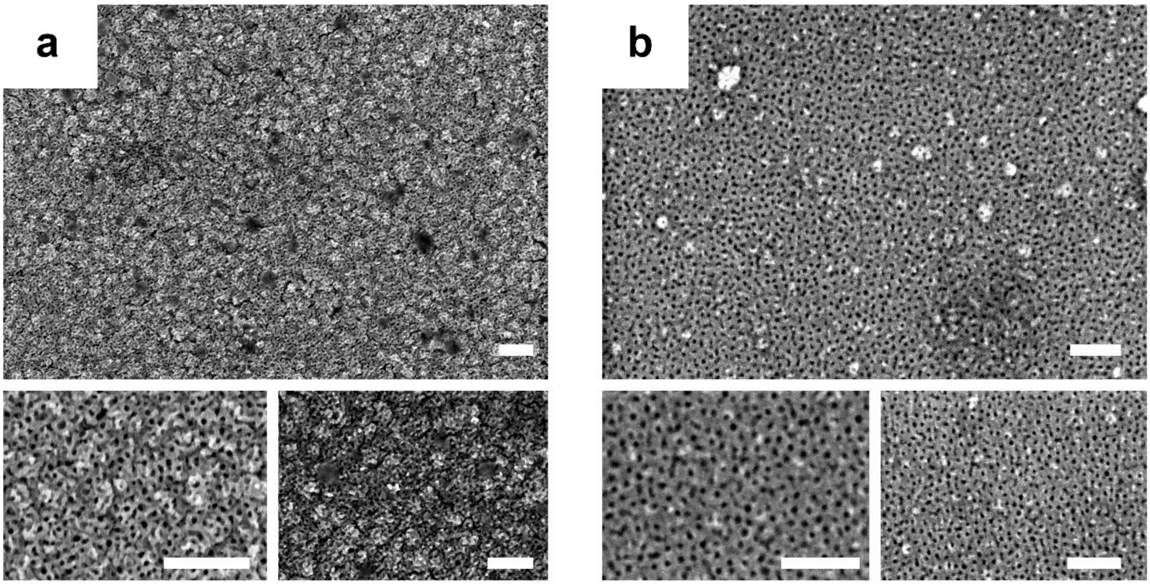

Figure S4. FE-SEM micrographs at different magnifications of the deposits obtained using Ni–Pt (bath 4) solution on (a) FTO-coated glass and (b) Si/Ti/Au substrates at −1.2 V (vs. Ag | AgCl) after circulating 7.5 C cm^{-2}. Scale bar: 300 nm.

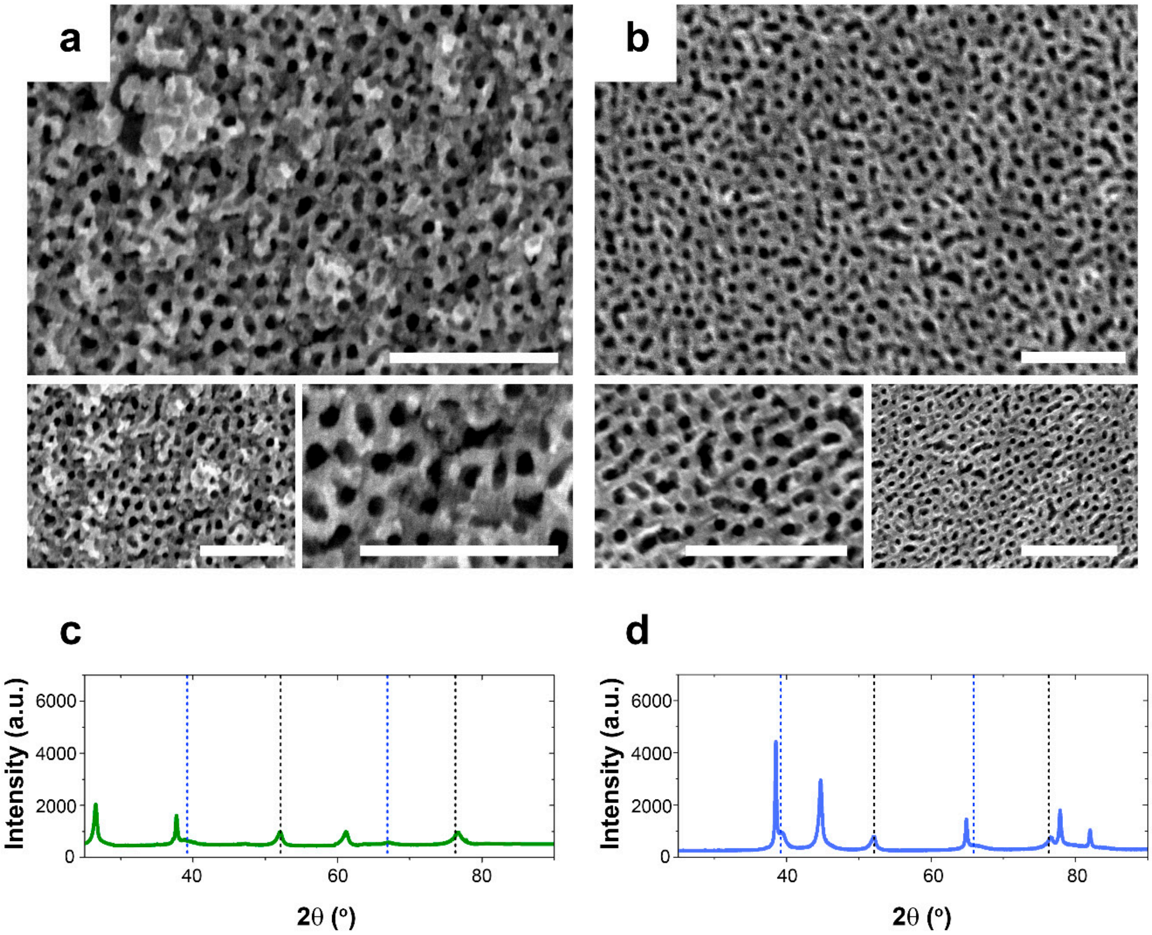

Figure S5. (**a**,**b**) FE-SEM micrographs at different magnifications and (**c**,**d**) XRD patterns of the deposits obtained using Ni–Pt (Bath 4) solution on (**a**,**c**) FTO-coated glass and (**b**,**d**) Si/Ti/Au substrates at −1.3 V (vs. Ag|AgCl) after 1000 cycling experiments. Scale bar: 300 nm.

© 2020 by the authors. Licensee MDPI, Basel, Switzerland. This article is an open access article distributed under the terms and conditions of the Creative Commons Attribution (CC BY) license (http://creativecommons.org/licenses/by/4.0/).
